# Studying the mechanism of sperm DNA damage caused by folate deficiency

**DOI:** 10.1111/jcmm.17119

**Published:** 2021-12-24

**Authors:** Wei Wang, Meilin Peng, Hongfang Yuan, Chunyan Liu, Yuan Zhang, Yiwei Fang, Yufang Su, Xinzong Zhang, Huiping Zhang, Yunge Tang, Kai Zhao

**Affiliations:** ^1^ Institute of Reproductive Health Tongji Medical College Huazhong University of Science and Technology Wuhan China; ^2^ NHC Key Laboratory of Male Reproduction and Genetics Guangdong Provincial Reproductive Science Institute(Guangdong Provincial Fertility Hospital) Guangdong China

**Keywords:** DNA double‐strand break, folic acid deficiency, sperm DNA injury, Rad54, reduced representation bisulphite sequencing

## Abstract

Sperm DNA injury is one of the common causes of male infertility. Folic acid deficiency would increase the methylation level of the important genes, including those involved in DNA double‐strand break (DSB) repair pathway. In the early stages, we analysed the correlation between seminal plasma folic acid concentration and semen parameters in 157 infertility patients and 91 sperm donor volunteers, and found that there was a significant negative correlation between seminal folic acid concentration and sperm DNA Fragmentation Index (DFI; *r* = −0.495, *p* < 0.01). Then through reduced representation bisulphite sequencing, global DNA methylation of sperm of patients in the low folic acid group and the high folic acid group was analysed, it was found that the methylation level in Rad54 promoter region increased in the folic acid deficiency group compared with the normal folic acid group. Meanwhile, the results of animal model and spermatocyte line (GC‐2) also found that folic acid deficiency can increase the methylation level in Rad54 promoter region, increased sperm DFI in mice, increased the expression of γ‐H2AX, that is, DNA injury marker protein, and increased sensitivity of GC‐2 to external damage and stimulation. The study indicates that the expression of Rad54 is downregulated by folic acid deficiency via DNA methylation. This may be one of the mechanisms of sperm DNA damage caused by folate deficiency.

## 1 INTRODUCTION

Infertility affects approximately 186 million people worldwide.^1^ Male factors account for approximately 35%–50% of the aetiology of infertile couples,^2^ and sperm DNA damage is an important factor affecting male infertility.^3^ For the past decade, sperm DNA Fragmentation Index (DFI) has been used to evaluate sperm DNA damage.^4^, ^5^ A high sperm DFI could cause male infertility and is closely related to recurrent spontaneous abortion.^6^ Therefore, reducing sperm DFI to improve male fertility is an urgent clinical problem that must be addressed.

In clinical practice, sperm DFI is commonly used to indicate the severity of sperm DNA damage. Elevated DFI seriously affects the pregnancy rate in ICSI cycles.^7^ In addition, sperm DFI is not strongly correlated with conventional semen parameters. Therefore, a sperm DNA fragmentation assay should be performed as an additional step in diagnosing male fertility.^8^ However, only a few studies were conducted on how to reduce the DFI of male sperm and the mechanism underlying DFI increase.

Sperm DNA damage is caused by many factors, including genetics, environment or their interaction,^9^, ^10^, ^11^ but the specific mechanism is unclear. It has been found that environmental factors can regulate the methylation of spermatogenesis‐related genes, which leads to sperm DNA damage.^12^ With abnormal miRNA expression found in patients with high sperm DFI, further mechanism research was conducted in mice.^13^, ^14^ All these findings indicate that epigenetic factors are one of the mechanisms leading to sperm DNA damage.

Diet, such as folic acid, is key in determining and maintaining sperm function, male fertility and normal reproductive system development.^15^ Antifolic acid drugs cause poor semen quality in treating various malignancies,^16^, ^17^, ^18^ but the mechanism is unclear. Growing evidence confirmed that folic acid is essential for male reproductive health.^19^ For example, Lambrot^20^ reported that lifelong folic acid deficiency delays the initiation of spermatocyte meiosis in C57BL/6 mice. Swayne^21^ and Ly^22^ observed that folic acid deficiency reduces testicular sperm production in BALB/c mice. Najafipour^23^ found that males with dietary folic acid below the recommended threshold show relatively low sperm density. Animal studies confirmed that the intraperitoneal injection of methotrexate, a folic acid antagonist, reduced the sperm density in the mice,^24^ whereas high doses of folic acid can competitively reverse the inhibiting effect of methotrexate on sperm production.^25^ But limited research was conducted on how folic acid regulates semen quality.

Folic acid is involved in the synthesis of important substances such as DNA, RNA and protein. Folic acid can generate S‐adenosylmethionine (SAM) through one‐carbon cycle metabolism. SAM is the main methyl donor in DNA methylation and participates in the methylation reaction in vivo.^26^, ^27^ In our previous study, the folic acid concentration was detected in the seminal plasma of 269 infertile male patients. Routine semen analysis was also conducted. The results showed that the sperm density in the seminal plasma group with low folic acid was significantly lower than that in the normal folic acid group. The mRNA and protein expression levels of three spermatogenesis‐related genes Esr1, Cav1 and Elavl1 were inhibited in the folic acid‐deficient mouse model.^28^ We detected the methylation levels of Esr1 Cav1 and Elavl1 promoter regions in the sperm of infertile patients. However, the overall methylation levels within these promoter sequences of these three genes were not significant between the folic acid‐deficient group and the normal folic acid group.^28^

γ‐H2AX protein expressed in the early stage of DNA damage and can reflect the degree of DNA damage.^29^ Therefore, DNA damage can be reflected by detecting γ‐H2AX.

The purpose of this study was to further explore the correlation between folic acid concentration in seminal plasma and semen parameters and the underlying mechanism. To complete the study, we analysed the correlation between seminal plasma folic acid and semen parameters from population samples, genome‐wide methylation detection was performed on sperm genomic DNA through reduced representation bisulphite sequencing (RRBS), and animal and cell models were then designed for verification.

## 2 MATERIALS AND METHODS

### 2.1 Study subjects and cell lines

The subjects were recruited from male infertile patients who visited the reproductive medicine centre of Tongji Medical College of Huazhong University of Science and Technology from March 2015 to August 2016 and from healthy volunteers donating sperm in the Hubei human sperm bank. After physical examination, the following information from the subjects was obtained through questionnaire: age, body mass index (BMI), ethnicity, medical history, alcohol and cigarette use, abstinence length and vitamin supplements. Participants who used alcohol, cigarettes and vitamins or had varicocele were excluded to avoid the effects of lifestyle factors on sperm DFI.

GC‐2 line was established by stable co‐transfection of freshly isolated mouse spermatocytes having the SV40 large T antigen gene and a temperature‐sensitive mutant of the p53 tumour suppressor gene, and GC‐2 cells were cultured in high‐glucose Dulbecco's Modified Eagle's Medium (Gibco) containing 10% foetal bovine serum (Gibco).

### 2.2 Reduced representation bisulphite sequencing (RRBS) detection

Twenty subjects from the low and normal folic acid groups [low concentration: 15.83 (11.27–17.08) nmol/L, *n* = 10, normal concentration: 26.07 (24.34–33.66) nmol/L, *n* = 10] with no differences in age (low folic acid group: 32 ± 4.2 years, normal folic acid group: 33 ± 3.4 years) and BMI (low folic acid group: 23.6 ± 4.3, normal folic acid group: 23.1 ± 5.5) were randomly selected to study the underlying mechanisms. The general characteristics of the selected subjects are shown in Table S1. Human sperm DNA was prepared following our previously reported optimized method.^30^ The obtained sperm DNA was segmented by MspI enzyme, and then purified. The purified fragments were repaired at the end, and the 3'end was added with A tail or added methylated joint and so on. Fragment size was selected through agarose gel electrophoresis, and a 230–380‐bp DNA fragment (including a 100‐bp connector) was selected. Bisulphite (heavy sulphite) treatment was performed, and PCR amplification was conducted to form a sequencing library. Illumina HiSeqTM 2500 was employed for the sequencing of the library with qualified quality control.

### 2.3 Bisulphite sequencing (BSP) for gene promoter DNA methylation

In brief, 2 µg of genomic DNA was treated with EpiTect bisulphite kit (#59104, Qiagen) to assay the DNA methylation levels of Rad54 promoters in human and mouse sperm. A PCR instrument (Life Technologies) was used to amplify the bisulphited DNA through bisulphite sequencing. The BSP primers were designed by Methyl Primer Express Software and are listed in Table S2. The PCR products were isolated with 2% agarose gel electrophoresis and purified using a DNA gel extraction kit (Beyotime Technology). The purified DNA was cloned into the PMD18‐T vector (Takara). Thirty positive clones from each sample were randomly selected for sequencing. The sequencing results were analysed using QUMA software on the website http://quma.cdb.riken.jp/. DNA methylation levels were calculated based on the percentage of the methylated CpG sites divided by the total CpG sites in the promoter region.

### 2.4 Mice rearing and dietary formula

Inbred C57BL/6 mice (female: 6 weeks old, *n* = 36; males: 6 weeks old, *n* = 18) were purchased from Hubei provincial centre for disease control and prevention (Wuhan, China). All mice were housed in cages under standard conditions (22°C on a 12 L:12 D cycle). Experimental diets (folic acid deficient, FD and folic acid sufficient, FS) were purchased from Beijing HFK Bioscience Co. Ltd. (No.11003800008778). Dietary formula was based on the guidelines of Lambrot.^20^ The mice in the folic acid‐deficient and folic acid‐supplemented sired groups were fed with FD (0.3 mg of folic acid per kg of body weight, *n* = 12) and FS diets (20 mg of folic acid per kg of body weight, *n* = 12) for 2 weeks and then mated with nonexperimental C57BL/6 males. The experimental group was maintained on FD and FS diets throughout pregnancy and lactation to generate male mice respectively. From weaning at Post 28 Days, the males of the next generation (F1) were given the same experimental food as their mothers until they were sacrificed. The normal group (marked as FN groups) and male mice were fed regular mouse chow.

### 2.5 Subject's semen collection and related parameters' detection

After a recommended period of sexual abstinence (2–7 days), the semen samples were collected by masturbation into a wide‐mouthed sterile plastic container in a separate room. The semen samples were marked with the subject's name and immediately delivered to a laboratory for testing. After liquefying in a water bath at 37°C, a conventional semen analysis was conducted using a computer‐assisted semen analysis system (SCA2000, Microptic) in accordance with the guidelines of the World Health Organization (2010). All samples were analysed by two trained laboratory technicians using the same instrument. Folate concentration of serum and seminal plasma were tested by electrochemical reaction.

### 2.6 Detection of serum folate concentration in mice

Serum folic acid levels of mice were measured as previously described to verify the effectiveness of the mouse model.^31^ Before killing mice, heart blood was collected, serum was obtained and folic acid concentration was measured. Folic acid was detected using the method of Boxmeer et al.^32^

### 2.7 Sperm DFI analysis

Sperm chromatin structure assay (SCSA) was performed to obtain sperm DNA fragmentation, as described in a previous study.^33^ Under acidic conditions, actinide orange (AO) combined with single‐stranded DNA produces red or yellow fluorescence and double‐stranded DNA produced green fluorescence. The two fluorescence types were detected by flow cytometry, and the ratio was calculated. In brief, the semen sample was diluted with TNE buffer and prepared into a sample with sperm concentration of 1 x 10^6^ spermatozoa/ml. The sperm suspension was treated with an acid detergent solution (0.15 M NaCl, 0.1% Triton X‐100 and 0.08 N HCl, pH = 1.2) for 30 s and then stained with 6 mg/L acridine orange (AO; Sigma‐Aldrich). Sperm integrity was analyzed by flow cytometry (Coulter Epics XL; 4. Beckman Coulter). Fluorescence images of 5000 sperms were collected, and each sperm sample was independently examined twice. DNA fragment index (DFI) was calculated from the DFI frequency histogram, which was obtained from the ratio of red fluorescence to total fluorescence intensity, and each sperm sample was independently examined twice.

### 2.8 Cell culture and treatment

Adherent GC‐2 (ATCC) cells were incubated at 37°C in a humidified incubator with 5% carbon dioxide. The cells were cultured in folic acid‐free (0 ng/ml), normal (4 ng/ml), folic acid (100 ng/ml) and folic acid (200 ng/ml) media for 6 days. Then total RNA, genomic DNA and total protein were extracted.

### 2.9 RNA extraction and quantitative real‐time PCR

Total RNA was extracted from the GC‐2 cells using TRIzol reagent (Invitrogen), and total RNA concentration was determined using a NanoDrop 2000 (Thermo Fisher Scientific). Reverse transcription was performed in accordance with the instruction of the first‐strand cDNA synthesis kit (Fermentas). The Table S2 shows the specific primers for Rad54 and β‐actin. qPCR was conducted on KAPA SYBR FAST qPCR Kit Master Mix (2X) ABI PrismTM (KAPA, KR0390) as previously described.^32^ Relative mRNA expression was calculated using 2^−∆∆CT^ method.

### 2.10 Gene expression detection by Western Blotting

Protein concentration was determined using the BCA Protein Assay Kit (Thermo‐Fisher) in accordance with the manufacturer's protocol, followed by 12% sodium dodecyl sulphate‐polyacrylamide gel electrophoresis separation. The protein was electro transferred onto a polyvinylidene fluoride membrane, which was blocked by using a blocking buffer containing 20 mM Tris‐HCl (pH 7.4), 150 mM NaCl and 0.1% (v/v) Tween 20 (TTBS) plus 5% fat‐free milk (w/v) for 2 h. The sample was incubated with a primary antibody (1:2000; primary antibody against γ‐H2AX:Abcam, ab26350; primary antibody against Rad54:Abbkine, ABP56756; primary antibody against GAPDH, ab103) at 4°C overnight and with a secondary antibody (horseradish peroxidase‐labelled goat anti‐rabbit or goat anti‐mouse antibody 1:5000) for 2 h. The results were obtained using the enhanced chemiluminescence (ECL) kit. Photographs were captured using a gel image analysis system.

### 2.11 γ‐H2AX, Rad54 and Rad51 protein staining and immunofluorescence test

The GC‐2 cells were cultured in media with folic acid concentrations of 0, 4, 100 and 200 ng/ml and then seeded in 6‐well dishes. After the GC‐2 cells covered 50% of the slides, the medium was replaced with PBS with a mass concentration of 5% H_2_O_2_. After 10 min on ice, the PBS was replaced with their respective medium. After 6 h in an incubator, cell climbing tablets were fixed with 100% cold methanol at −20°C for 15 min and permeabilized with 0.2% Triton X‐100 for 10 min. The cell climbing tablets were incubated overnight at 4°C with γ‐H2AX, Rad54 and Rad51 protein monoclonal antibody (1:500, Abcam, ab26350; Abbkine, ABP56756; Abcam, ab88572). After being washed three times, the cell climbing tablets were incubated with the secondary antibody conjugated with Alexa Fluor 488 (Molecular Probes) for 2 h at room temperature. DAPI was then used for staining. Images were acquired using OlymPus BX53 fluorescence microscope.

### 2.12 Immunofluorescence analysis of cryosections of testis

Immunodetection of γ‐H2AX and Rad54 was performed as previously described.^34^ The cryosections of testes were obtained after fixation in 4% phosphate‐buffered formaldehyde for 24 h, permeabilized with 0.2% Triton X‐100 for 10 min, blocked for 1 h with 5% normal goat serum in TBS with 1% BSA and 0.2% Triton X‐100, and finally incubated overnight at 4°C with γ‐H2AX monoclonal antibody (1:500, Abcam, ab26350) and Rad54 antibody (Abbkine, ABP56756). After being washed three times, the cells were incubated with the secondary antibody conjugated with Alexa Fluor 488 (Molecular Probes) for 1 h at room temperature. DAPI was used for staining. Images were acquired using OlymPus BX53 fluorescence microscope.

### 2.13 Sperm count and sperm DFI detection in mouse epididymal cauda

C57 mice were killed, and their bilateral epididymis was quickly isolated. In each of the caudal of the epididymis, three mouths were cut. The epididymis was then placed in 1 ml F10 incubation solution, incubated for 10 min and then fully and evenly mixed. Volume of 100–900 µL diluent was diluted 10 times (diluent in 37°C water bath incubation in advance). Finally, the concentration of sperm in the EP tube was measured with the haemocytometer, and the sperm reserve of caudal epididymal was determined.^35^ Motility was expressed as percentage motile forms.^36^ The detection steps of sperm DFI were the same as above.

### 2.14 Haematoxylin and eosin staining

The animals were killed with chloral hydrate, and the testis was fixed in 10% formalin for 24 h and was then embedded in paraffin. The coronal sections (5 µm thick) were set on poly‐l‐lysine‐coated slides, stained with haematoxylin and eosin and observed under a microscope.

### 2.15 Statistical analysis

All data were analysed using Statistical Package for the Social Sciences (SPSS) software (version 24.0, SPSS, Inc.). Results were expressed as median (range) or mean ± SD as appropriate. Data were tested for normality and homogeneity of variance. For intergroup difference test, *t* test was used for the data subjected to normality and homogeneity of variance. Otherwise, the nonparametric test was employed. *p* < 0.05 was statistically significant. Data with * *p* < 0.05, ** *p* < 0.01 and ***p < 0.005 were considered to have statistical difference and significant statistical difference. WB protein band was analysed by Image J software.

## 3 RESULTS

### 3.1 Relationship between the concentration of seminal plasma folic acid and sperm DNA fragmentation index (DFI) in infertile patients

**FIGURE 1:**
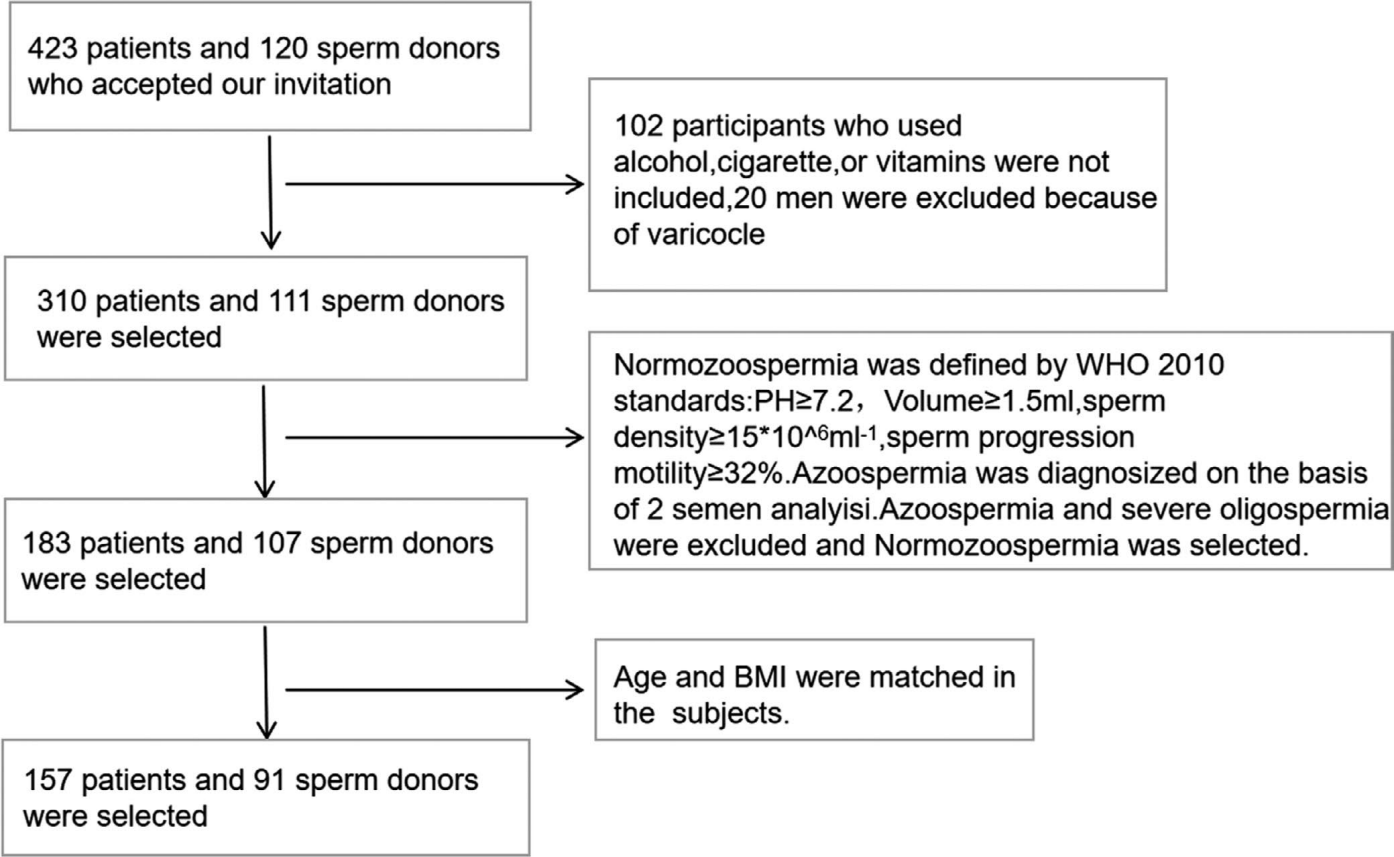
Flow chart of the screening of subjects in this study

We selected 248 subjects from 423 infertile patients and 120 sperm donor volunteers. The screening process is shown in Figure 1. Among the 248 subjects, 91 were sperm donors and 157 were infertility patients. The general characteristics of the subjects were shown in Table S3. Then, the semen parameters were logarithmically converted, and multiple linear regression was used to analyse the correlation between the folic acid concentration of seminal plasma and semen parameters. Following the adjustment for age and BMI, the folic acid concentration of seminal plasma was negatively correlated with sperm DFI (*r* = −0.495, *p* < 0.01), but was not correlated with other semen parameters.

### 3.2 Sperm genome methylation for low and normal folic acid groups

**FIGURE 2:**
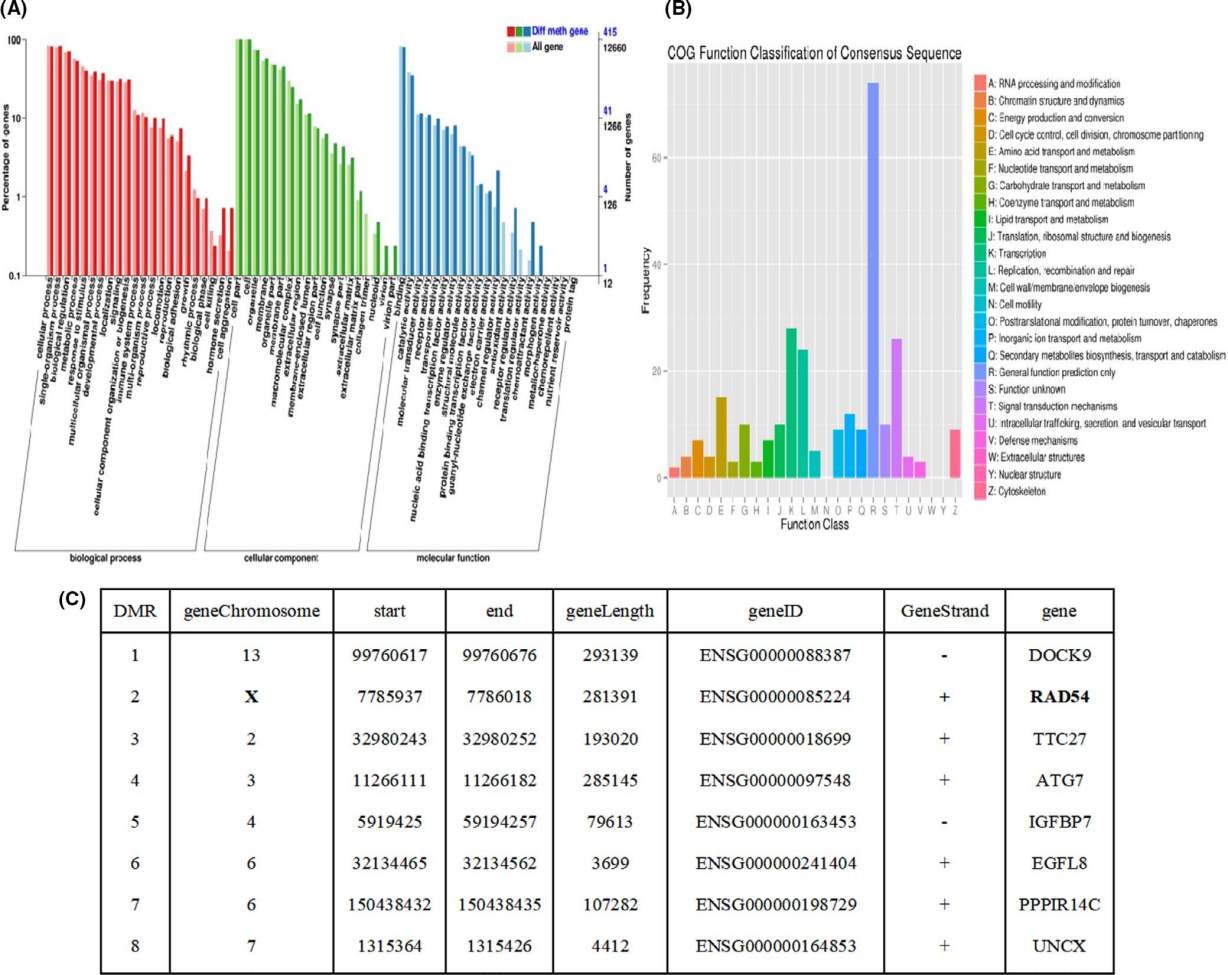
GO and COG annotation analysis of DMR‐associated genes. In (A) the x‐coordinate represents the contents of each GO classification, the left y‐coordinate represents the percentage of the number of genes and the right y‐coordinate represents the number of genes. In (B) the x‐coordinate represents the content of COG classification, and the y‐coordinate represents the number of genes. (C) represents significantly different DNA methylation regions and the associated gene annotations

Genome‐wide DNA methylation of sperm DNA was assessed by RRBS. Bioinformatics analysis revealed 1287 regions of differential methylation (DMRs) in the sperm genomes of the two groups. Compared with the normal folic acid group, the DMRs in the low folic acid group increased decreased 823 and these DMRs related genes were further analysed by cluster analysis using GO. As shown in Figure 2A, the genes related to DMRs focused on cell metabolism, biological regulation, immune response, cell development, substance metabolism, damage repair, apoptosis and signalling pathway function. Direct homology classification analysis (COG) was performed on the gene products, and the results are shown in Figure 2B. As shown in Figure 2C, the DMR‐related genes were compared with the GO, KEGG functional database by BLAST and the annotation of these genes was obtained to analyse the gene function. Finally, the Rad54 gene intrigued us because it is closely related to DNA double‐stranded break repair (DSB).

### 3.3 Effects of folic acid on C57BL/6 mouse

**FIGURE 3:**
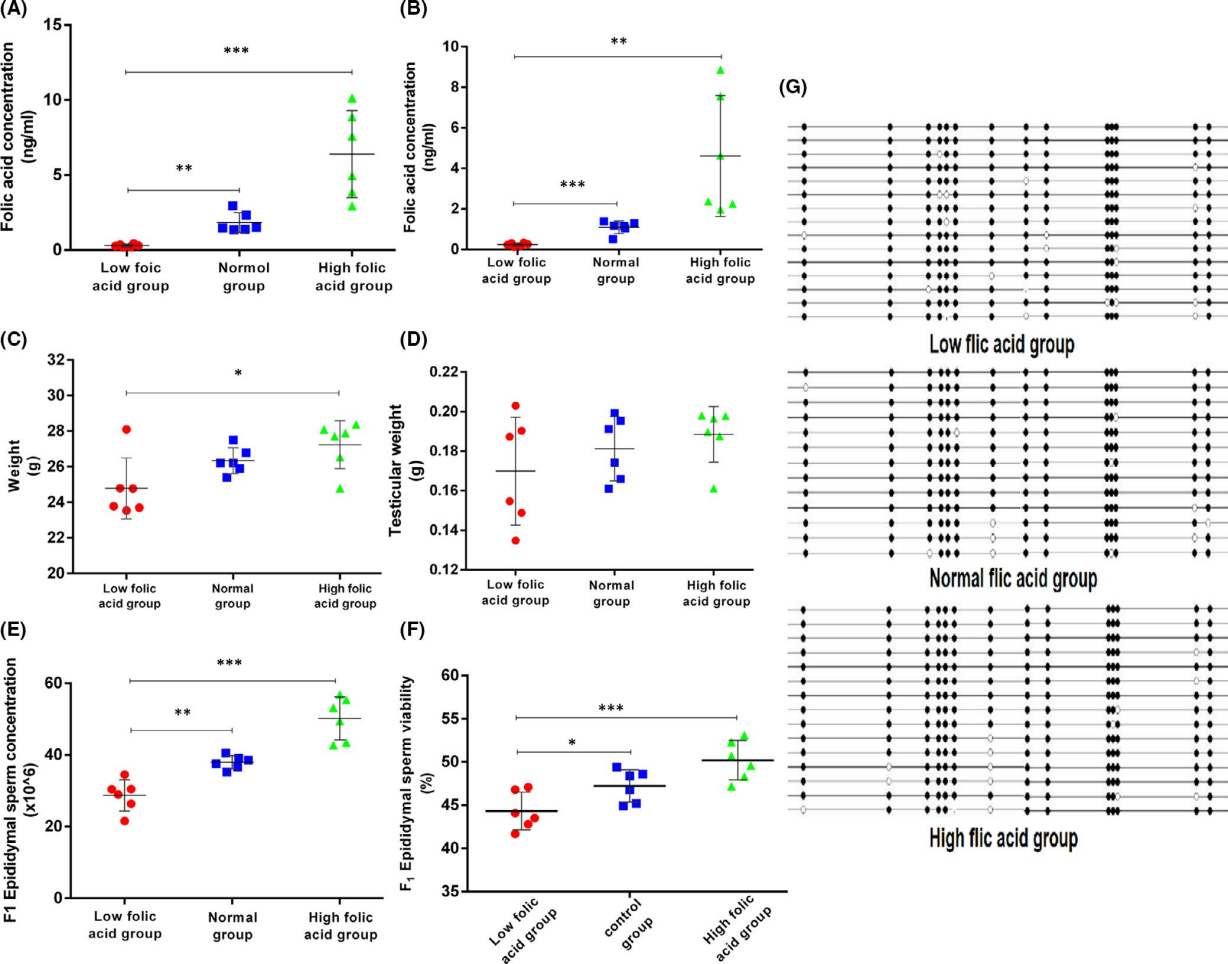
Animal experiment‐related results. (A and B) represent the serum folic acid concentration of parental and progeny respectively. (C and D) represent the weight of F1 generation and testicular weight respectively. (E) shows the percentage of sperm in the tail of the epididymis. (F) is the concentration of sperm in the tail of epididymis. (G) shows the methylation results of the PROMOTER region of Rad54 gene of sperm DNA of F1 generation mice. The white dots represent the methylation sites, and the black dots represent the nonmethylation sites. The data are presented as mean ± SD, bars indicate SD, *n* = 6, *p < 0.05, **p < 0.01, *** *p* < 0.005

The serum folic acid concentration in parental (F0) female mice and 8‐week‐old F1 male mice was measured to determine whether the models were established successfully. The results show that the models were established successfully (Figure 3A,B). The folic acid concentration of F0 in the FD group was significantly lower than that in the FS and FN groups (Figure 3A). The folic acid concentration of F1 in the FD group was significantly lower than that in the FS and FN (*p* < 0.005) groups (Figure 3B). Figure 3C shows that the weights of F1 mouse of the FD and FN groups were not statistically different (*p* > 0.05) but were significantly lower than that of the FS group (*p* < 0.05). Figure 3D shows no difference in testicular weight among the three groups. Figure 3E,F reveal that the epididymal sperm concentration and viability in the FD group were significantly lower than those in the FS and FN groups.

**FIGURE 4:**
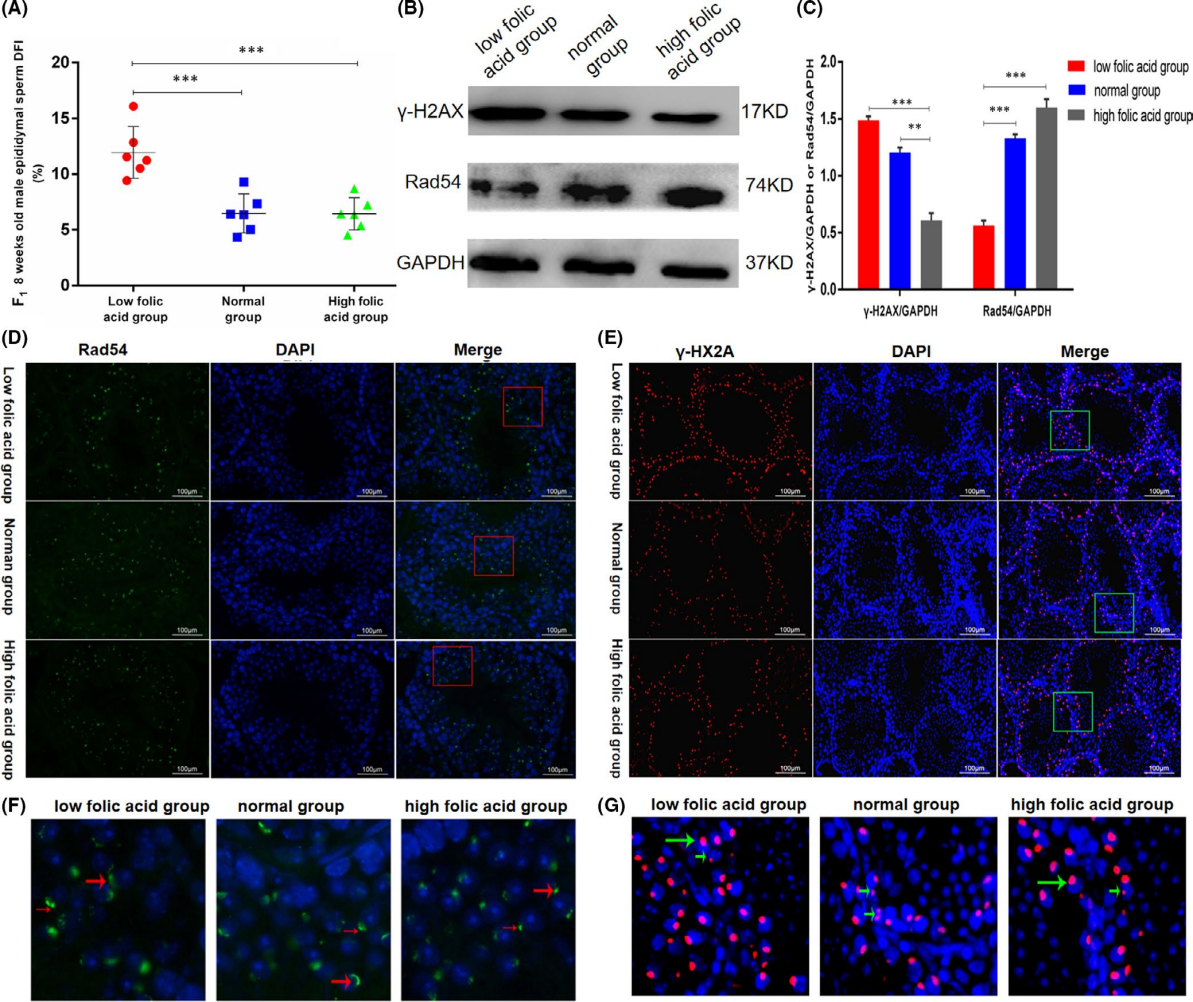
Effect of folic acid deficiency on sperm integrity and related protein expression. (A) represents epididymal spermatozoa DFI of mice aged 8 weeks in F1 generation; (B) represents Western blot results; (C) represents the grey value of the protein bands. (D) represents immunofluorescence results of Rad54 protein; (F) is the red box amplification result; The red arrow represents the location and expression of Rad54 protein in testicular tissue. (E) shows the immunofluorescence of gamma‐H2ax protein and (G) shows its green box magnification. The green arrow represents the location and expression of γ ‐H2AX protein in testicular tissue. The data are presented as mean ± SD, bars indicate SD, *n* = 6, *** *p *< 0.005

Epididymal sperm DFI was detected by sperm chromatin structure analysis (SCSA) to explore the effect of folic acid deficiency on sperm genomic DNA integrity. Figure 4A shows that the sperm DFI of 8‐week‐old mice in the FD group was significantly higher than those in the FS (*p* < 0.005) and FN groups (*p* < 0.005).

### 3.4 Folic acid affects the expression of Rad54 and γ‐H2AX proteins

Three mice aged 12 weeks were randomly selected from each group to detect the expression of Rad54 and γ‐H2AX in testicles by using immunofluorescence. The results are shown in Figure 4D,E respectively. Figure 4F,G are the enlarged images of the area in the box in Figure 4D,E respectively. The red arrow and green arrows represent the localization of Rad54 protein and γ‐H2AX protein on the nucleus respectively. The results show that the FD group had significantly lower Rad54 expression but higher γ‐H2AX expression compared with the FS and FN groups. Figure 4B represents Western blot results, Figure 4C represents the grey value of the protein bands. The trend of Western blot results was consistent with that of immunofluorescence (Figure 4B,C), indicating that folic acid deficiency in seminal plasma affects the expression of Rad54 gene, thus increasing the sperm DFI in epididymal sperm and making the testes vulnerable to environmental damage and stimulation during spermatogenesis.

### 3.5 Folic acid deficiency affects the methylation of Rad54 gene promoter region

**FIGURE 5:**
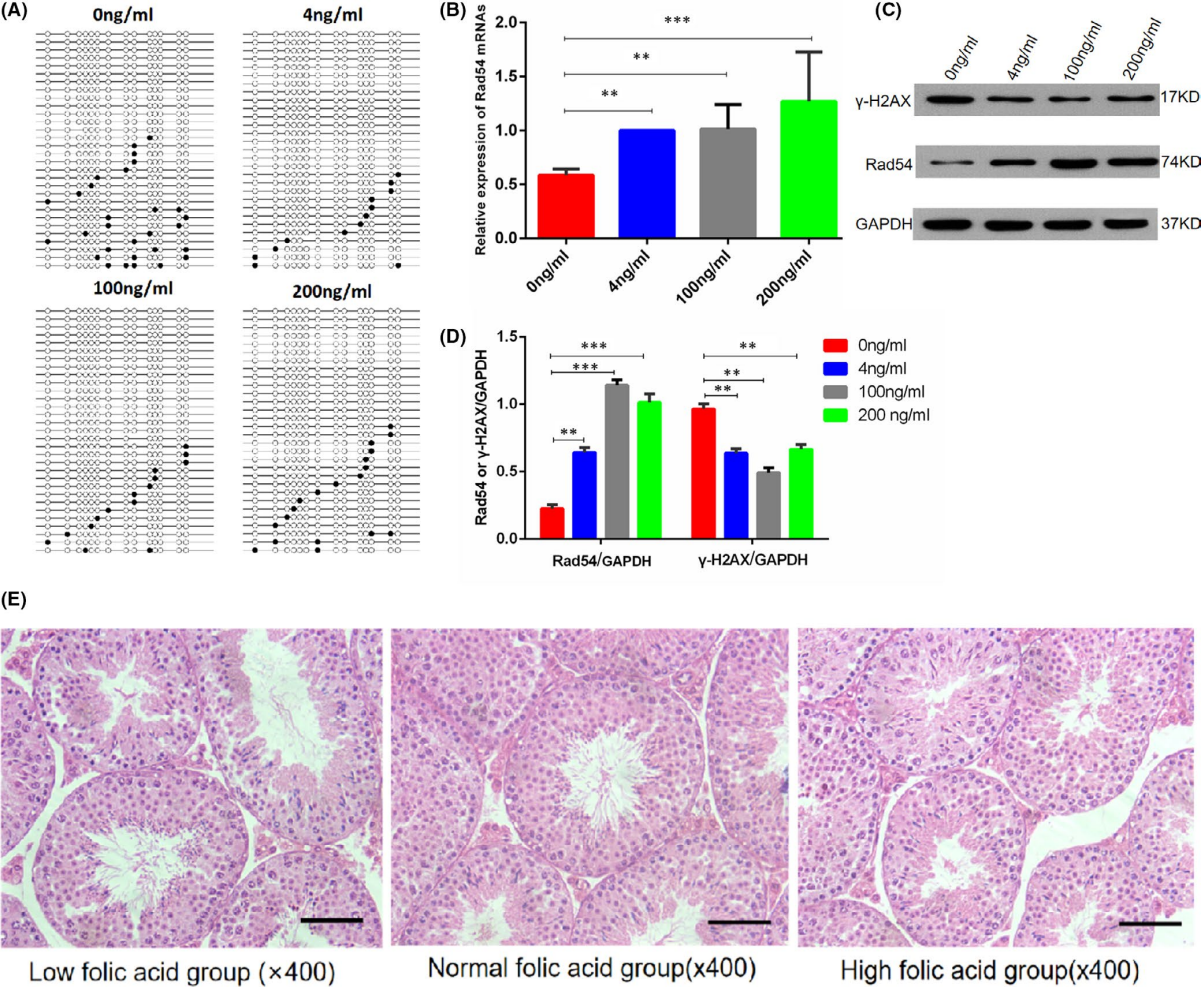
Cell test results and haematoxylin and eosin staining results of testes of F1 generation mice. (A) is a ball‐and‐stick model for CpG site methylation analysis. White dots represent sites that have not been methylated, while black dots represent sites that have been methylated. (B, C and D) show the influence of folic acid on the expression of Rad54 and γ‐H2AX without external injury stimulation respectively. (B) represents the mRNA expression of Rad54 gene, (C) represents the expression of Rad54 and γ‐H2AX protein and (D) represents the grey value of the protein bands. (E) shows the results of haematoxylin and eosin staining of testes in F1 generation mice. The data are presented as mean ± SD, bars indicate SD, *n* = 6; Bar size is 50 µm; *p < 0.05, **p < 0.01, *** *p* < 0.005

Genomic DNA was extracted from four cell line models with different folic acid concentrations, and the methylation status of each CpG site in Rad54 gene promoter region was quantitatively assessed by BSP. Thirty clones randomly selected from each group were sequenced, and 14 CpG sites were detected. Figure 5A shows that the methylation frequency of CpG site in Rad54 gene promoter region was 10.4% in the 0 ng/ml folic acid group, 3.2% in the 4 ng/ml group, 3.5% in the 100 ng/ml group and 4.5% in the 200 ng/ml group. The methylation level of CpG sites in Rad54 gene promoter regions was higher in the folic acid‐free group than in the folic acid‐supplemented group, indicating that folic acid deficiency could affect the methylation statue of Rad54 gene promoter. In addition, genomic DNA was extracted from mouse sperm, and the above method was used to detect the methylation level of Rad54 gene promoter region. The methylation frequency of CpG site in Rad54 gene promoter region was 10.9% in the FD group, 6.4% in the FN group and 6.6% in the FS group (Figure 3G).

Figure 5B‐D show the influence of folic acid on the expression of Rad54 and γ‐H2AX without external injury stimulation respectively. Figure 5B represents the mRNA expression of Rad54 gene, the results showed that the expression of Rad54 gene in the 0 ng/ml folic acid group was significantly lower than that in the 4 ng/ml, 100 ng/ml and 200 ng/ml folic acid concentration groups. Figure 5C represents the expression of Rad54 and γ‐H2AX protein, and Figure 5D represents the grey value of the protein bands, and the expression trend of Rad54 protein was consistent with that of Rad54 gene. Figure 5E shows the results of haematoxylin and eosin staining of testes in F1 generation mice, however, haematoxylin and eosin staining results of the testis showed no significant difference.

### 3.6 Folic acid deficiency affects the expression of Rad54 gene and increases cell damage

**FIGURE 6:**
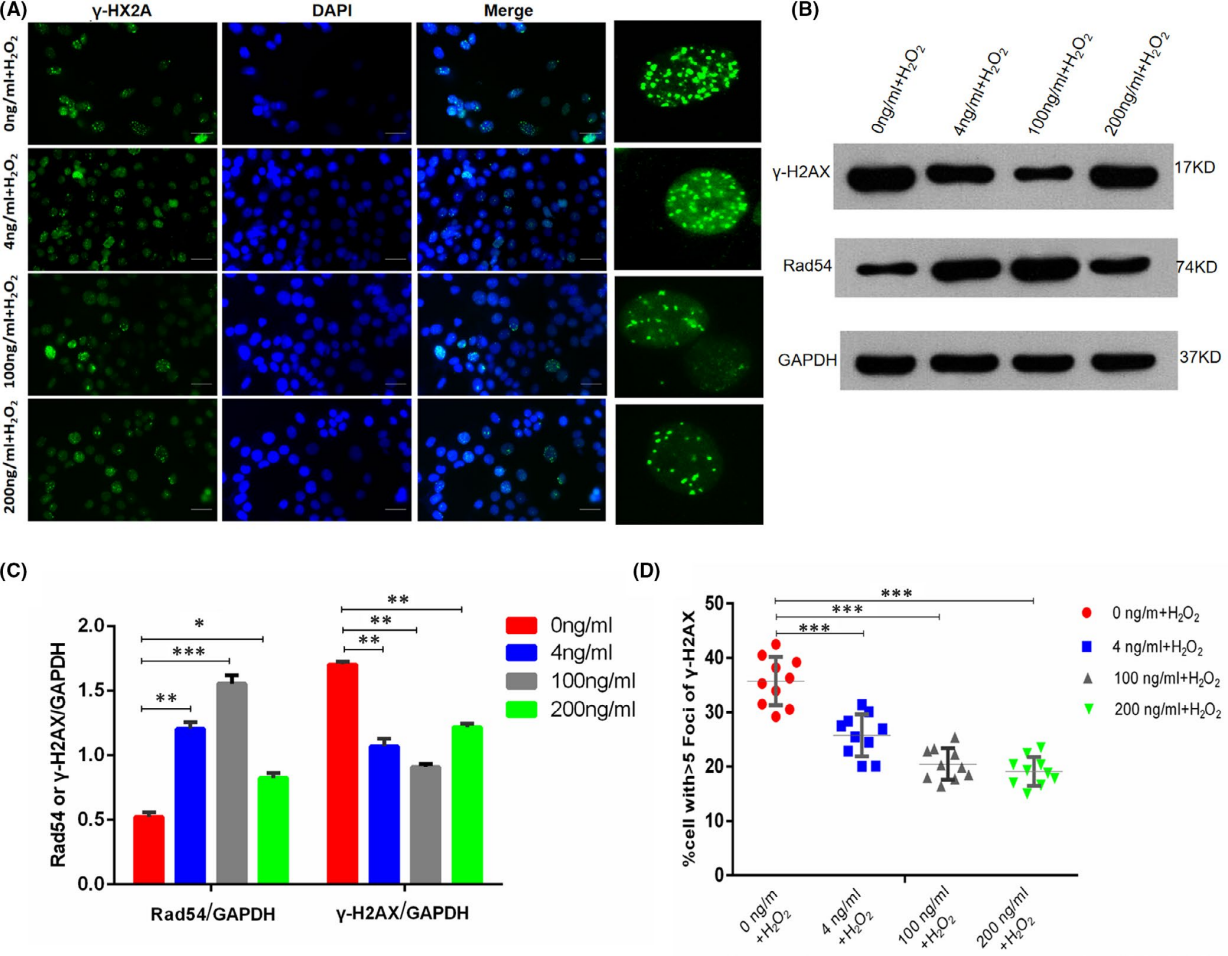
Expression of DNA damage markers in GC‐2 cells after external injury stimulation. (A) shows the expression of γ‐H2AX in GC‐2 cells, and (D) shows the percentage of GC‐2 cells with more than five fluorescence foci. (B and C) show the expression of Rad54 and γ‐H2AX protein and the grayscale of the protein bands after the addition of H_2_O_2_ respectively. The data are presented as mean ± SD, bars indicate SD, *n* = 10; Bar size is 100 µm; *p < 0.05, **p < 0.01, ***p < 0.005

γ‐H2AX expression was detected by immunofluorescence, 10 fields were randomly selected for each group under 200‐fold microscope field of view, and the percentage of cells with more than five fluorescence focal points in each field was counted. Statistical results showed that the folic acid concentration in the 0 ng/ml folic acid group was significantly higher than those in the 4 ng/ml (*p* < 0.005), 100 ng/ml (*p* < 0.005) and 200 ng/ml (*p* < 0.005) folic acid concentration groups (Figure 6A,D). Figure 6B,C show the expression of Rad54 and γ‐H2AX protein and the grayscale of the protein bands after the addition of H_2_O_2_ respectively. The results showed that the γ‐H2AX protein expression in the folic acid concentration in the 0 ng/ml folic acid group was significantly higher than those in the 4, 100 and 200 ng/ml folic acid concentration groups. The expression trend of Rad54 protein was opposite to that of γ‐H2AX protein.

**FIGURE 7:**
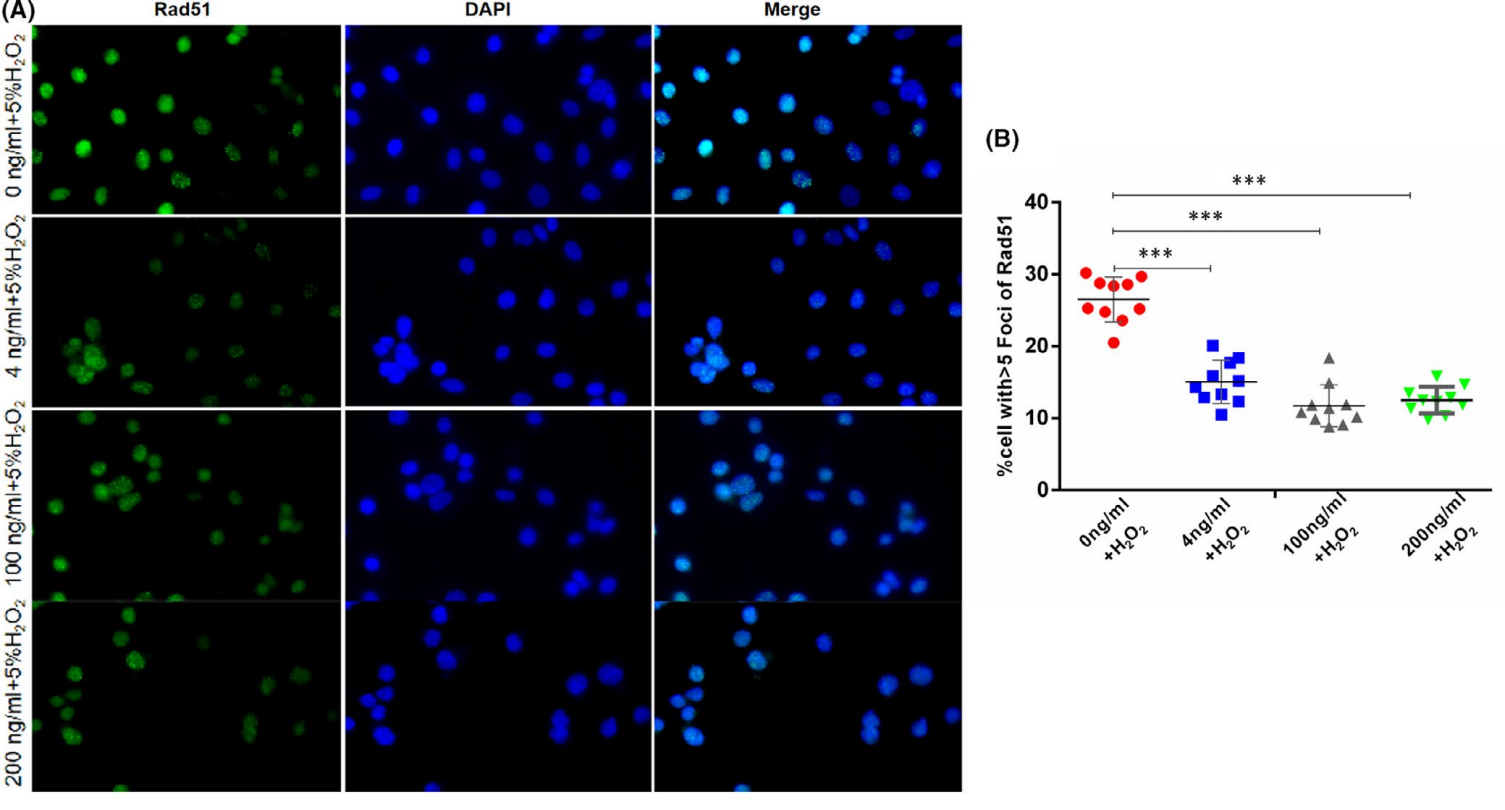
Immunofluorescence of Rad51 protein in GC‐2 cells after external injury stimulation. (a) shows the expression of Rad51 protein in GC‐2 cells, and (B) shows the percentage of cells with A fluorescence focus of Rad51 protein greater than 5 in GC‐2 cells. The data are presented as mean ± SD, bars indicate SD, *n* = 10; Bar size is 100 µm; ***p < 0.005

Rad51 protein, which can also reflect the changes of DNA damage sites, was also detected using the same method. Immunofluorescence results in Figure 7A and statistical findings in Figure 7B show that the Rad51 protein expression in the folic acid‐free group was significantly higher than those in the 4, 100 and 200 ng/ml groups. The expression levels of γ‐H2AX and Rad54 protein were detected by WB, and the results are shown in Figure 6B,C. Immunofluorescence and WB findings revealed that the GC‐2 cells in the folic acid‐free group were highly susceptible to external damage.

## 4 DISCUSSION

Sperm DNA damage is a major cause of defective sperm function and infertility^37^ and is linked to decreased pregnancy rates of natural fertilization, intrauterine insemination and in vitro fertilization.^38^ Assessment of sperm DNA damage is a better independent predictor of male fertility than traditional semen analysis.^5^ A high level of sperm DNA damage indicates low sperm count and motility or abnormal sperm morphology.^39^, ^40^, ^41^

Our clinical study found that the concentration of folic acid in the seminal plasma of infertile men was lower than that of normal men. Sperm DFI detection revealed that the concentration of folic acid in the seminal plasma of infertile men was statistically significantly lower than that in men with low DFI. Hence, a significant negative correlation occurs between seminal plasma folic acid and sperm DFI. This finding is consistent with the research of Boxmeer et al.^42^ Animal studies also showed that folic acid deficiency could seriously increase DFI percentage (folic acid deficiency group vs control group, 5.0 ± 0.9 vs. 2.6 ± 0.1, *p* < 0.05).^21^ The current study is the first to analyse the correlation between human seminal plasma folic acid level and sperm DFI using a large population sample and highlight the importance of folic acid to male reproductive health.

Spermatogenesis is a complex process of biochemical and morphological changes, including mitosis, meiosis and sperm deformation and release, all involving the synthesis of DNA, RNA and various proteins and the regulation of methylation. Folic acid also plays a role in these processes and provides the methylated donor SAM for DNA methylation.^43^ Therefore, the mechanism of sperm damage caused by low folic acid must be studied from an epigenetic perspective.

High‐throughput methylation sequencing analysis revealed differences in the methylation of Rad54 gene between the low‐folic acid groups and normal groups. The methylation level in the Rad54 gene promoter region was increased in the low‐folic acid group. Rad54 gene is involved in DNA double bond break (DSB) repair, and Rad51 and Rad54 proteins are involved in the HR repair of DNA double strand breaks, the key to maintaining genomic stability.^44^, ^45^, ^46^ Rad54 protein is a SWI2/SNF2 family member and a key player in HR pathway and the deposition and stabilization of Rad51 foci at double strand breaks (DSBs).^47^ Rad54 participates in chromatin remodelling and promotes Rad51 dissociation at the end of HR.^48^ In addition, experimental studies on animals and cells have confirmed that low folic acid can increase spermatocyte damage. Our result also revealed that low folic acid increases the sensitivity of GC‐2 cell lines to injury stimulation. These results support our previous hypothesis that low folic acid increases the methylation level in the Rad54 promoter region and consequently decreases the HR repair efficiency of DNA double strand break.

Low folic acid could also reduce epididymal sperm concentration and sperm motility in F1 mice. Our results are consistent with the study of Ly et al.^22^ but inconsistent with that of Lambrot et al.^20^ The discrepancy may be due to the different samples and sampling times used for sperm counts. Lambrot et al.^20^ used frozen epididymis from mice aged 15 weeks. In the current research, fresh epididymis from mice aged 8 weeks were used. Meanwhile, folic acid deficiency seriously increased the rate of testicular DNA double strand break damage, which is consistent with the study of Kelly et al.^49^ An increased methylation level in the Rad54 promoter region was found in the GC‐2 cell line in folic acid‐free group, with significant increases in some sites.

Rad51 protein, which is also a marker protein DNA double‐stranded injury, was also detected by immunofluorescence. Rad51 protein is mainly involved in the HR repair of double‐stranded injury, and γ‐H2AX protein is involved in the whole process of DSB.^50^, ^51^, ^52^ Here, Rad51 protein was significantly higher in the folic acid‐free group than in the folic acid supplementation group, thus further verifying that DSB repair pathway was inhibited.

## 5 CONCLUSIONS

Our research suggest that low seminal plasma folic acid affects the methylation level of the Rad54 promoter region and consequently the expression of Rad54 protein, leading to an increase in sperm DFI and the sensitivity of spermatogenesis to external injury stimulation. This study also provides a mechanistic reference for clinical folic acid supplementation for men to reduce sperm DNA damage.

## CONFLICT OF INTERESTS

The authors declare no conflicts of interest.

## AUTHOR CONTRIBUTIONS

**Wei Wang:** Data curation (lead); Investigation (lead); Writing – original draft (lead); Writing – review & editing (lead). **Meilin Peng:** Data curation (equal); Formal analysis (equal); Writing – original draft (equal); Writing – review & editing (equal). **Hongfang Yuan:** Conceptualization (equal); Formal analysis (equal). **Chunyan Liu:** Conceptualization (equal); Formal analysis (equal). **Yuan Zhang:** Data curation (equal); Software (equal). **Yiwei Fang:** Data curation (equal); Software (equal). **Yufang Shu:** Conceptualization (equal); Software (equal). **Xinzong Zhang:** Conceptualization (equal); Funding acquisition (equal). **Huiping Zhang:** Conceptualization (lead); Funding acquisition (lead). **Yunge Tang:** Conceptualization (lead); Data curation (lead); Funding acquisition (lead). **Kai Zhao:** Formal analysis (lead); Funding acquisition (lead); Methodology (lead); Project administration (lead); Writing – original draft (lead); Writing – review & editing (lead).

## INFORMED CONSENT

All participants provided an informed written consent.

## Supporting information

Table S1

Table S2

Table S3

## Data Availability

The data that support the findings of this study are available from the corresponding author upon reasonable request.
