# Supplementary material for: Studying the mechanism of sperm DNA damage caused by folate deficiency
Source: J Cell Mol Med. 2021 Dec 24;26(3):776–88. doi: 10.1111/jcmm.17119 (PMC8817123; doi:10.1111/jcmm.17119)
Supplement: Supplementary file 1 — Table S1 [file JCMM-26-776-s001.doc]

**Supplemental Table 1** General characteristics of the 20 subjects used in the RRBS experiment

| Characteriteristics | Low folate(n=10)^a^ | Normal folate(n=10)^a^ | *P*-value^b^ |
| --- | --- | --- | --- |
| *Demgraphic* |  |  |  |
| Age(y) | 32(25-39) | 33(26-40) | 0.85 |
| BMI | 23.6(22.6-24.0) | 23.1(19.2-27.5) | 0.68 |
| *Semen parameters* |  |  |  |
| Ejaculate volumc(ml) | 3.4(2.7-5.0) | 3.6(2.5-5.8) | 0.57 |
| Sperm density(10^6/ml) | 55.87(19.07-354.40) | 182.11(57.03-127.08) | 0.00 |
| Sperm count(10^6） | 160.31(61.01-354.40) | 214.57(129.63-498.09) | 0.01 |
| Sperm progressive motility(%) | 46.84(32.39-65.37) | 46.77(35.98-64.13) | 0.76 |
| Spermnomal morphology(%) | 43.85(32.33-61.23) | 46.34(28.94-50.24) | 0.83 |
| Duration of abstrention(d) | 5(3-7) | 5(3-7) | 0.59 |
| Seminal plasma folate | 15.83(11.27-17.08) | 26.07(24.34-33.66) | 0.00 |

^a^ All variables were presented as median (range);

^b^ Differences between Low folate group and Normal folate group were analyzed using the independent sample T test.

Abbreviation: BMI: Body Mass Idex.
