# Supplementary material for: Studying the mechanism of sperm DNA damage caused by folate deficiency
Source: J Cell Mol Med. 2021 Dec 24;26(3):776–88. doi: 10.1111/jcmm.17119 (PMC8817123; doi:10.1111/jcmm.17119)
Supplement: Supplementary file 2 — Table S2 [file JCMM-26-776-s003.doc]

**Supplemental Table 2** qPCR primers and methylation primers

| name | sequence |
| --- | --- |
| *Rad54*  *β-Actin* | F: TTGATAATTTGGGATGGGGA  R: GGAAGCCCTTGACTCAGCTA |
|  | F: CTGAGAGGGAAATCGTGCGT  R: CCACAGGATTCCATACCCAAGA |
| BSP-Rad54-F  BSP-Rad54-R | F: TAGTGGTTTGTTGAGTTTTTG  R: AAAACCAATTAAAAATCCAAAT |
