# Supplementary material for: Studying the mechanism of sperm DNA damage caused by folate deficiency
Source: J Cell Mol Med. 2021 Dec 24;26(3):776–88. doi: 10.1111/jcmm.17119 (PMC8817123; doi:10.1111/jcmm.17119)
Supplement: Supplementary file 3 — Table S3 [file JCMM-26-776-s002.doc]

**Supplemental Table 3** General characteristics of subjects

| characteriteristic | Infertile patients(n=157)^a^ | Sperm donor volunteer(n=91)^a^ | P-value^b^ |
| --- | --- | --- | --- |
| *Demgraphic* |  |  |  |
| Age(y) | 30(26-35) | 31(26-37) | 0.06 |
| BMI(kg/m^2^) | 21.3(17.3-29.7) | 22.1(17.2-29.4) | 0.23 |
| *Semen parameters* |  |  |  |
| Ejaculate volumc(ml) | 2.9(1-5.9) | 3.5(1.5-5.5) | <0.01 |
| Sperm density(10^6/ml) | 63.2(15.29-242) | 71.50(15.67-388.6) | <0.01 |
| Sperm count(10^6) | 189.28(33.13-1400.1) | 255.25(39.46-1998.09) | <0.01 |
| Sperm progressive motility (%) | 42.31(33.1-76.58) | 46.55(32.01-81.57) | 0.37 |
| Sperm nomal morphology(%) | 41.21(29.2-51.2) | 42.77(29.2-53.5) | 0.26 |
| pH | 7.4(6.0-7.4) | 7.4(7.2-7.5) | 0.00 |
| Duration of abstrention(d) | 4(2-7) | 5(2-7) | 0.24 |
| DFI | 29.35(7.11-49.63) | 25.77(5.68-41.04) | <0.01 |

^a^ All variables were presented as median (range);

^b^ Differences between Infertile patients group and Sperm donor volunteer group were analyzed using the independent sample T test.

Abbreviation: BMI: Body Mass Idex.
